# The Flip Side of Distractibility—Executive Dysfunction in Functional Movement Disorders

**DOI:** 10.3389/fneur.2020.00969

**Published:** 2020-09-11

**Authors:** Anne-Catherine M. L. Huys, Kailash P. Bhatia, Mark J. Edwards, Patrick Haggard

**Affiliations:** ^1^Department of Clinical and Movement Neurosciences, Queen Square Institute of Neurology, University College London, London, United Kingdom; ^2^Neuroscience Research Centre, Institute of Molecular and Cell Sciences, St George's University of London, London, United Kingdom; ^3^Institute of Cognitive Neuroscience, University College London, London, United Kingdom

**Keywords:** attention, attention network test, functional neurological disorder, functional movement disorders, conversion disorder, movement disorders, executive, cognitive

## Abstract

Attention plays a crucial role in functional neurological disorders. Attention to the symptoms leads to their exacerbation and distraction to their improvement or even transitory disappearance.

**Objective:** The aim was to test if the alerting, orienting and particularly the executive aspect of attention are affected in functional movement disorders.

**Methods:** Thirty patients with a functional movement disorder, 30 patients with an organic movement disorder and 30 healthy controls performed the attention network test.

**Results:** The alerting and orienting effects were normal, but executive control of attention under conflict was abnormal in patients with functional movement disorders, compared to patients with an organic movement disorder and healthy controls.

**Conclusion:** Executive dysfunction seems to be an important secondary feature of functional movement disorders, due to the overutilization of attentional resources for explicit movement control. Furthermore, it provides an explanation for seemingly unrelated symptoms commonly associated with functional movement disorders, such as concentration difficulties and fatigue.

## Introduction

Functional movement disorders (FMD) manifest as movement disorders that are genuine, but often illogical or incongruent. As such they often fluctuate, and non-physiological maneuvers may improve or worsen them. They cannot be explained by any structural, biochemical or genetic abnormality. Functional disorders are known by many different names: in psychiatry they are known as conversion disorders. The idea being that psychological trauma is converted into physical symptoms. The terms psychogenic or psychosomatic share the same rationale. Other names are medically unexplained or non-organic disorders.

Attention can be viewed as a set of processes that allow us to ignore irrelevant information and use the brain's limited processing capacity for the information that is most important in a given situation (1).

Attention plays a pivotal role in functional movement disorders. This is apparent in terms of symptoms, signs, and neurobiology: attention to the symptoms leads to their exacerbation and distraction to their improvement or even transitory disappearance (2). Indeed, distractibility is one of the main diagnostic features of functional movement disorders (3, 4): functional tremor, for example, diminishes or disappears transitorily when the patient's attention is distracted away from it. Hoover's sign in functional leg weakness is the demonstration of normal strength when the movement is performed automatically, without any attention, as opposed to weakness or paralysis when the person attempts to move the affected limb voluntarily. At a pathophysiological level, abnormally focused attention can be implicated in symptom generation (5). In a Bayesian framework, attention to the symptom, coupled with a strong prior belief in its presence, can override the actual sensory input and shift the overall perception and/or execution to conform to the symptom (6, 7).

A crucial question is therefore whether attention is abnormal in FMD. Standard measures of attention have generally been found to be normal in functional movement disorders and even in functional neurological disorders in general, with the exception of non-epileptic attack disorder (8, 9). Yet, in the case of functional movement disorders, relatively few studies have been performed to date.

Recent models analyze attention into three separable components, dependent on different brain networks: alerting, orienting and executive. The alerting network, as the name implies, leads to alertness and vigilance. The orienting network allows to focus either on a location in space or a modality, i.e. a specific feature. Finally, the executive network is involved in “top-down” control, in focal attention, and conflict resolution (withholding a response in favor of a less obvious response) (10, 11). The attention network test (ANT), is a behavioral test that allows to differentiate the alerting, orienting and executive networks' efficiencies in a single task (12). The ANT involves responding to a left or right target arrow by pressing a left or right keyboard key as quickly as possible. The target arrow is surrounded by two flankers on either side: lines (neutral flankers), or arrows pointing in the same direction (congruent flankers) or the opposite direction with regards to the target arrow (incongruent flankers). Prior to the presentation of the arrow there is either no cue, a temporally informative cue, or a temporally and spatially informative cue. The alerting effect is measured by the reaction time (RT) difference between the temporally informative cue condition and the no cue condition. The orienting effect is the RT difference between the both spatially and temporally informative cue condition and the solely temporally informative cue condition. The executive or conflict effect is the difference in RT to the incongruent vs. the congruent flanker arrows.

A previous ANT study in patients with chronic fatigue syndrome with depression and without depression and a healthy control group, showed normal alerting and orienting effects and a non-significant trend to abnormalities in the executive component in both patient groups (13).

In a modified version of the ANT, the ANT-I (14), the alerting cue is a tone presented before the visual cue, the cue validity is only 50%, i.e., non-informative and there are only two flanker types (congruent and incongruent arrows). A previous study using ANT-I with fibromyalgia patients showed an impairment of both the alerting and the executive components of attention (15). Pain, the main feature of fibromyalgia, is well-known to influence attention.

Given the clinically evident central role of attention in functional movement disorders, we set out to systematically evaluate the different attentional networks in people with functional movement disorders by means of the ANT. So as to differentiate the impact of a functional movement disorder from a movement disorder per se, people with FMD were compared not only to healthy controls, but also to people with an organic movement disorder. Other than through the influence of medication or fatigue, the *alerting* effect was not expected to be affected in functional movement disorders. Although a strong focus on the symptom might lead to an inability to shift the attentional focus away from it, a sudden exogenous stimulus in space automatically attracts attention (so called “bottom-up” attention). The orienting aspect of attention in the attention network test was therefore expected to be normal. *Executive* function is the most cognitively demanding and non-automatic aspect of attention. Out of the three components it is the most likely to be affected in functional movement disorders.

## Methods

### Participants

Patients with functional and organic movement disorders were primarily recruited from the clinical practice of expert movement disorder and functional movement disorder specialists (KB and ME) and the diagnosis was confirmed on the day of the study by a further neurologist (ACH). The characteristics of the 30 patients with a functional movement disorder (FMD), the 30 patients with an organic movement disorder (organic controls OC) and the 30 healthy controls (HC) are summarized in Table 1.

**Table 1:** Study participant characteristics.

	**Healthy control (*n* = 30)**	**Organic movement disorder (*n* = 30)**	**Functional movement disorder (*n* = 30)**
**M:F**	14:16	15:15	13:17
**Age** (range)	44.7 y (24–79 y)	48.0 y (21–77 y)	47.5 y (21–79 y)
**Movement disorder**	None: 30	Action tremor • upper limb: 22 • head: 8 • voice:5 Weakness: 1 Dystonia • cervical: 13 • segmental: 2 2 • hands/writer's cramp: 5 • oromandibular:1 • laryngeal: 4 Myoclonus: 1	Functional action tremor • upper limb: 24 • lower limb: 6 • head: 2 • palate: 1 Functional weakness: 6 Functional dystonia: 6 Functional gait disorder: 5 Paroxysmal FMD: 4 Functional myoclonus: 1 Functional stiffness: 2
**Medication** (daily medication that may affect attention)	Antidepressants • SSRI: 2 • SSNRI: 1	Benzodiazepines: 3 Anticholinergics: 1 Antidepressants • SSRI: 3 • SSNRI: 1 Antiepileptics • pregabalin: 1	Benzodiazepines: 5 Antidepressants • SSRI: 6 • SSNRI: 2 • Tricyclic: 2 • Tetracyclic: 1 Antiepileptics • pregabalin/gapapentin: 8 • carbamazepine: 1 Opioids • non-morphine: 5 • morphine-like: 3 Neuroleptics: 2
**Anxiety** HADS—A sub-score (SD)	5.2 (3.5)	7.0 (3.6)	8.9 (4.6)
**Depression** HADS—D sub-score (SD)	2.4 (2.5)	3.7 (2.4)	8.0 (3.8)

*The organic action tremors were caused by dystonic tremor, essential tremor and Wilson's disease in one case. Note that 15 FMD patients and 27 organic controls had more than one movement disorder type and 11 FMD and 3 organic controls took more than one analgesic. “Functional gait disorder” comprised a functional gait disorder not explained by any other listed FMD type. SSRI, selective serotonin reuptake inhibitor; SSNRI, selective serotonin and noradrenaline reuptake inhibitor. HADS, Hospital Anxiety and Depression Scale (score ≤7: normal, 8–10: mild, 11–14: moderate, 15-−21 severe affection)*.

### Attention Network Test

**Figure 1:**
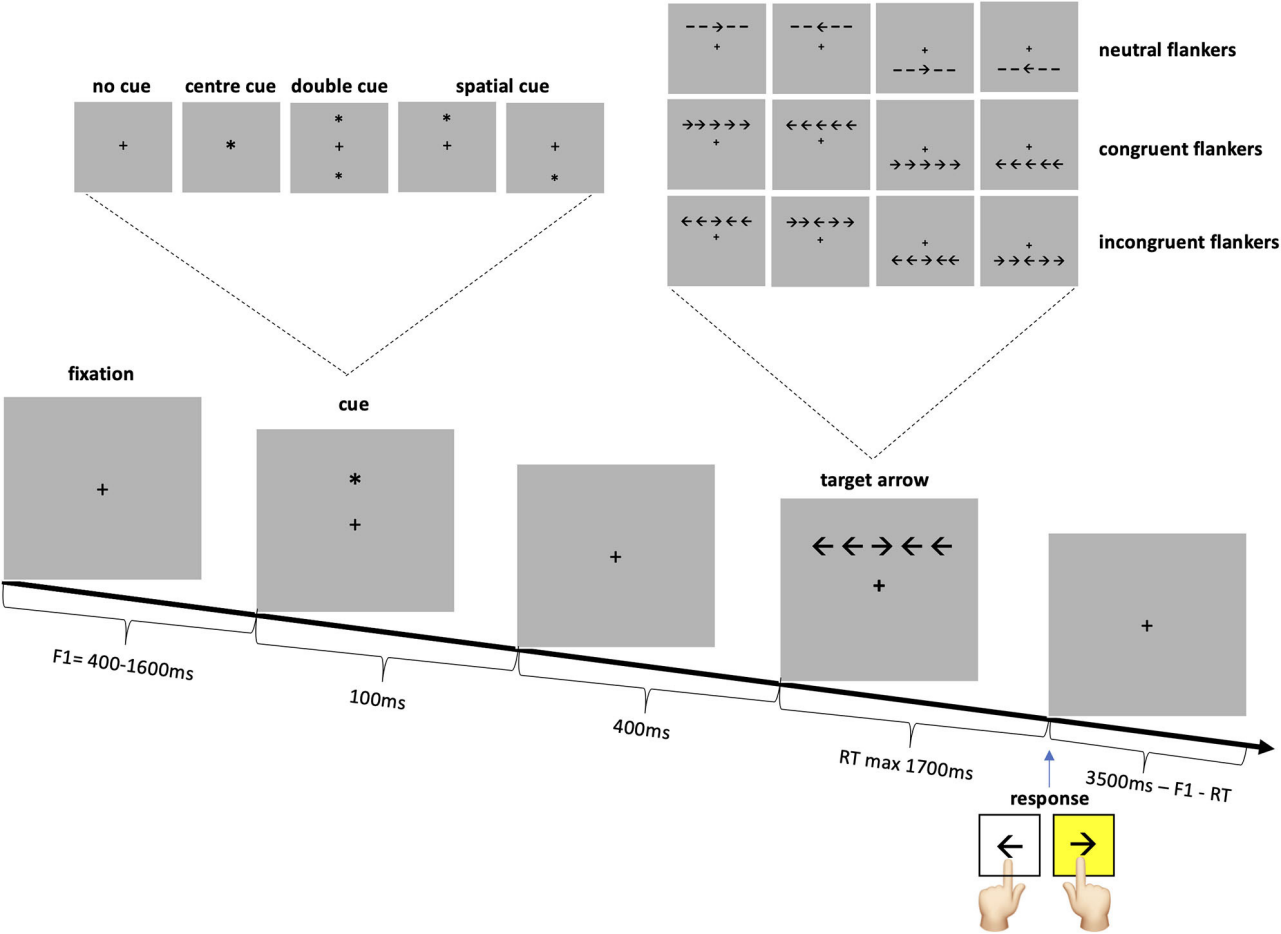
Attention network test experimental setup.

Participants performed the attention network test described in the original paper by Fan and colleagues (12). Subjects were seated at a viewing distance of 65 cm in front of a 19-inch computer screen, with a refresh rate of 75 Hz. The task was to respond as quickly as possible to a target arrow, presented slightly above or below the fixation point, pointing left or right, by pressing the corresponding keyboard key (Figure 1). The target arrow was surrounded by four flankers which were either congruent or incongruent arrows or neutral lines. The target was preceded by one of four cue conditions: no cue, center, double or spatial cue. Subjects were informed that the center, double and spatial cues were temporally informative, as they always preceded the arrow by 400 ms. The spatial cue additionally indicated the location of the arrow with 100% accuracy. Subjects were instructed to try to maintain fixation on the fixation cross at all times. Each cue condition was presented on ¼ and each flanker on 1/3 of the trials.

After 24 practice trials with feedback as to the correctness and the response speed, 288 trials were performed in three blocks. Stimuli were presented and the responses recorded using Matlab® R2015b (Mathworks, Natick, MA, USA) in conjunction with the Cogent 2000 toolbox (www.vislab.ucl.ac.uk/cogent.php). STATA® (StataCorp. 2013. Stata Statistical Software: Release 13. TX: StataCorp LP) was additionally used for data analysis.

### Analysis

Trials for which the reaction time was too slow (>1,700 ms), even after having been repeated once and incorrect trials were excluded from all analyses. Similar to previous literature, reaction times that fell more than 1.5 times the interquartile range above the 3rd or below the 1st quartile within each subject were excluded as outliers (HC: 2.8% of trials, OC: 2.4%, FMD 3.6%) (16, 17).

If the assumptions of the parametric tests were not met, the equivalent non-parametric test was performed. Since ANOVA is relatively robust to departures from normality (18) and the sample sizes were always equal, it was performed as long as the variances were not unequal. Multiple comparisons were corrected by the Šidák-Holm method; the uncorrected test statistic and effect size are shown, together with the corrected *p-*value. The significance level was set at 0.05, two-tailed.

## Results

### Age and Gender

The age between the three groups was not significantly different (one-way ANOVA [*F*_(2, 87)_ = 0.41, *p* = 0.67], nor was the male to female ratio (Pearson's chi-square χ^2^(2) = 0.27, *p* = 0.87).

### Error Rates

In terms of error rates there was no significant difference between the three groups for either flanker type (Kruskal–Wallis: congruent flankers (HC: *M* = 0.61%, SD = 1.08, OC: *M* = 0.28%, SD = 0.48, FMD: *M* = 0.36%, SD = 0.58): χ^2^(2) with ties = 0.99, *p* = 0.61, η^2^ = 0.012, neutral flankers (HC: *M* = 0.96%, SD = 1.30, OC: M = 0.71%, SD = 0.81, FMD: *M* = 0.67%, SD = 1.47): χ^2^(2) with ties = 1.97, *p* = 0.37, η^2^ = 0.0004, incongruent flankers (HC: *M* = 4.1%, SD = 3.6, OC: *M* = 3.2%, SD = 2.7, FMD: *M* = 3.8%, SD = 4.0): χ^2^(2) with ties = 0.81, *p* = 0.67, η^2^ = 0.014. Significantly more errors (pressed the wrong key) occurred with incongruent flankers (*M* = 3.71%, SD = 3.45) than with either congruent (*M* = 0.42, SD = 0.76) or neutral flankers (*M* = 0.78, SD = 1.22) [Kruskal–Wallis χ^2^(2) with ties = 107.7, η^2^ =0.40, *p* = 0.0001, Holm-Šidák corrected two-sample Wilcoxon rank-sum tests incongruent vs. congruent: *z*_uncorr_ = −9.26, *p*_corr_ < 0.001, incongruent vs. neutral *z*_uncorr_ = −8.11, *p*_corr_ < 0.001].

### Overall RT

FMD patients had significantly slower overall reaction times than either control group (Table 2) (one-way ANOVA [*F*_(2, 87)_ = 8.74, *p* = 0.0003, η^2^ = 0.17]; *post-hoc* two-sample *t*-tests with Šidák-Holm correction: FMD vs. HC *t*_uncorr_(58) = −3.81, *p*_corr_ = 0.001, *d* = −0.98, FMD vs. OC *t*_uncorr_(58) = −2.69, *p*_corr_ = 0.019, *d* = −0.69, HC vs. OC *t*_uncorr_(58) = −1.39, *p*_corr_ = 0.17, *d* = −0.36).

**Table 2:** Reaction times for the different cues, flanker types, and overall for the three groups.

	**Cue** RT in ms (SD)				**Flanker** RT in ms (SD)			**Overall RT in ms (SD)**
	**None**	**Center**	**Double**	**Spatial**	**Congruent**	**Neutral**	**Incongruent**	
**HC** (*n* = 30)	638.9 (94.6)	612.3 (99.0)	602.6 (92.4)	566.0 (97.5)	576.4 (90.7)	575.9 (92.0)	666.3 (106.0)	604.7 (95.0)
**OC** (*n* = 30)	674.9 (99.8)	651.2 (99.8)	636.8 (97.7)	595.2 (96.3)	606.8 (94.5)	605.2 (92.2)	709.8 (109.4)	639.3 (97.4)
**FMD** (*n* = 30)	754.6 (152.0)	738.7 (144.7)	721.4 (137.8)	684.9 (145.2)	685.0 (139.7)	683.3 (136.4)	812.3 (158.0)	724.7 (144.0)

*The group average reaction times are given in milliseconds, the standard deviations are in brackets. Note that for the cue types the different flanker types are collapsed together and vice versa*.

The fact that patients with FMD had overall slower reaction times canceled itself out in the alerting, orienting and conflict effects, since these effects were calculated by subtracting the reaction time with one type of cue or flanker from another.

### Alerting, Orienting, and Conflict Effects

As in the original paper, the alerting, orienting and conflict effects were calculated in the following manner for each subject:

$$\begin{array}{l} \text{Alerting effect}=\text{RT No cue}–\text{RT double cue}\\ \text{Orienting effect}=\text{RT center cue }–\text{RT spatial cue}\\ \text{Conflict effect}=\text{RT incongruent flanker}–\text{RT congruent flanker} \end{array}$$

Note that for the alerting effect the double cue was used as it is postulated that in both the “no cue” and the “double cue” conditions the attentional focus is large. Hence the only difference is the timing information. For the orienting effect, on the other hand, the control was the single central cue as it leads to attention to one location, similar to the spatial cue.

**Figure 2:**
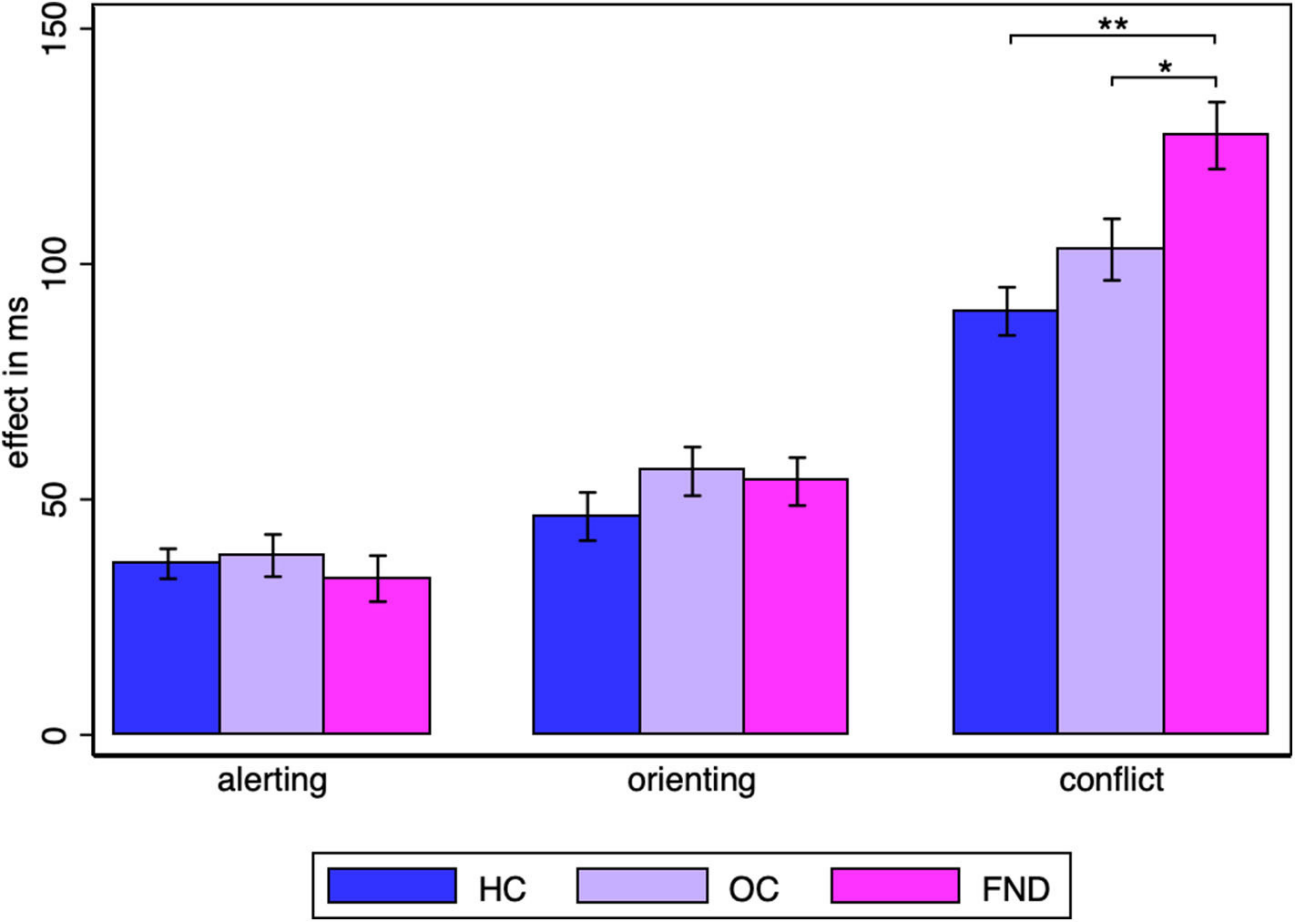
Alerting, orienting and conflict effect group averages. Statistically significant Šidák-Holm corrected results are marked by asterisks: * *p*_corr_ < 0.05, ** *p*_corr_ < 0.001. The standard error of the mean is shown by the error bars.

Since the question of interest is whether there is a difference between the three groups for each of the attentional networks, a separate one-way ANOVA was performed for each of the alerting, orienting and conflict effects. The group difference was not significant for the alerting (HC: *M* = 36.3, SD = 17.4, OC: *M* = 38.1, SD = 24.5, FMD: *M* = 33.2, SD = 26.8) [*F*_(2, 87)_ = 0.34, *p* = 0.71, η^2^ = 0.008], nor for the orienting effect (HC: *M* = 46.4, SD = 28.0, OC: *M* = 56.0, SD = 28.3, FMD: *M* = 53.8, SD = 27.9) [*F*_(2, 87)_ = 0.96, *p* = 0.39, η^2^ =0.022], but it was significant, with a large effect size for the conflict effect [*F*_(2, 87)_ = 9.03, *p* = 0.0003, η^2^ = 0.17] (Figure 2). Šidák-Holm corrected pairwise comparisons confirmed that there was a significant difference in the conflict effect between the FMD group (*M* = 127.3, SD = 38.9) and either control group (HC: *M* = 90.0, SD = 28.0, OC: *M* = 103.1, SD = 35.8) [FMD vs. HC two-sample *t*-tests *t*_uncorr_(58) = −4.27, *p*_corr_ < 0.0002, *d* = −1.1, FMD vs. OC Wilcoxon rank sum test: *z*_uncorr_ = −2.53, *p*_corr_ = 0.023, *r* = −0.33], but not between the two control groups (Wilcoxon rank sum test: *z*_uncorr_ = −1.21, *p*_corr_ = 0.23, *r* = −0.16).

The effects in the healthy controls were similar to those in the original paper by Fan et al., in which the alerting effect was 47 ms (SD = 18 ms), the orienting effect 51 ms (SD = 21) and the conflict effect 84 ms (SD = 25 ms) (12).

FMD patients had significantly higher anxiety scores on the HADS (Hospital Anxiety and Depression score) than healthy controls, but not than their organic counterparts (one-way ANOVA: [*F*_(2, 87)_ = 6.87, *p* = 0.0017, η^2^ = 0.14]; *post-hoc* Šidák corrected two-sample *t*-tests FMD vs. HC *t*_uncorr_(58) = −3.57, *p*_corr_ = 0.001, FMD vs. OC: *t*_uncorr_(58) = −1.82, *p*_corr_ = 0.17, HC vs. OC *t*_uncorr_(58) = −1.98, *p*_corr_ = 0.21). The depression scores were higher in the FMD group than in the OC group, with the latter having higher scores than the healthy controls (one-way ANOVA: [*F*_(2, 87)_ = 29.7, η^2^ = 0.41, *p* < 0.0001]; Šidák-Holm corrected two-sample Wilcoxon rank sum tests: FMD vs. HC *z*_uncorr_ = −5.12, *p* < 0.001, FMD vs. OC *z*_uncorr_ = −4.41, *p*_corr_ < 0.001, HC vs. OC *z*_uncorr_ = −2.36, *p*_corr_ = 0.018). The higher antidepressant use in the respective groups reflected these differences.

So as to exclude that the observed difference between the groups was caused by medications or additional medical conditions that might have affected attention, the analysis was repeated after all subjects on relevant daily medication (benzodiazepines, opioids, antiepileptics, antidepressants, neuroleptics, anticholinergics) were excluded. This also excluded subjects with chronic pain, depression or anxiety important enough to warrant medication. Analysis of the remaining 27 healthy controls, 23 organic controls and 14 functional movement disorders patients gave the same conclusion for the alerting, orienting and conflict effect. (Kruskal–Wallis test was not significant for the alerting effect (χ^2^(2) = 0.70, *p* = 0.70, η^2^ = 0.021), nor for the orienting effect (χ^2^(2) = 2.49, *p* = 0.29, η^2^ = 0.0080), but it was significant with a moderate to large effect size for the conflict effect (χ^2^(2) = 8.83, *p* = 0.012, η^2^ = 0.11). Šidák-Holm corrected, two-sample *t*-test confirmed that there was a significant difference between the FMD group (*M* = 127.1 ms, SD = 40.0) and the healthy controls (*M* = 89.1 ms, SD = 29.4) [*t*_uncorr_(39) = −3.47, *p*_corr_ = 0.0026, *d* = −1.14]. Similarly, Šidák-Holm corrected two-sample Wilcoxon rank-sum test gave a significant difference between the FMD group and the organic controls (*M* = 98.5 ms, SD = 32.0) (*z*_uncorr_ = −2.25, *p*_corr_ = 0.024, *r* = –0.37). The overall RT in all subjects not on any relevant medication showed a trend to a difference between the three groups (Kruskal–Wallis test: χ^2^(2) = 5.73, *p* = 0.057, η^2^ = 0.061), with Šidák-Holm corrected, two-sample *t*-tests showing a significant difference between the FMD group (*M* = 731.6 ms, SD = 175.2) and the healthy controls (*M* = 604.4 ms, SD = 97.8) [*t*_uncorr_(39) = −3.00, *p*_corr_ = 0.0094, *d* = −0.99] and between the FMD group and the organic controls (*M* = 629.2 ms, SD = 97.9), [*t*_uncorr_(35) = −2.29, *p*_corr_ = 0.028, *d* = −0.78].

Within each group, the reaction times with congruent and neutral flankers were virtually identical (Table 2). Thus, the group difference for the conflict effect was due to incongruent flankers leading to stronger inhibition in people with FMD rather than congruent flankers providing stronger facilitation.

### Side

Note that responses on the more affected side (which could include an ipsilateral body part other than the arm) in subjects with asymmetric symptoms (15 FMD patients, 25 OC patients), were not significantly slower in either group compared to the responses with the less affected side [one-sample *t*-test of the difference between the more and less affected side in FMD: *t*_(14)_ = 0.51, *p* = 0.62, in OC *t*_(24)_ = 0.96, *p* = 0.35].

## Discussion

We found a specific isolated abnormality in executive function in FMD, based on a standard test of attention, the ANT. This test measures three distinct components of visual attention, namely alerting (being prepared for an event occurring), orienting (shifting spatial processing resources to a specific location) and conflict processing (overcoming a response tendency triggered by distracting information). We found preserved orienting and alerting functions of the attentional system in a group of FMD patients. However, we observed an executive dysfunction related to conflict resolution in our FMD group, notably a failure to inhibit the effects of incongruent distractor information.

A possible limitation is that our movement disorder groups comprised a large number of people with upper limb tremor which might have directly interfered in their responses. The study could be repeated with even more varied groups or by using verbal responses (voice switch) rather than key presses. Nevertheless, it is very unlikely that the upper limb tremor in the FMD group could explain the current findings, since the movement disorder control group had a very similar number of upper limb tremors; the reaction times between the more and less symptomatic sides in people with asymmetric symptoms did not differ significantly in either group; and the three component effects of the ANT are computed as differences between reaction times. Thus, overall slower RTs in the FMD group would not affect the results. It is also evident that the observed executive dysfunction in FMD cannot be attributed to the simple presence of a movement disorder, or a disease in general, since we did not observe an equivalent effect in the organic movement disorders group. It is furthermore independent of medication use, pain and psychiatric comorbidities. The present study also suggested that the overall increased reaction times in the functional movement disorders group [see also (19, 20) for similar results] were largely independent of medication use, pain and psychiatric comorbidities. Fatigue, which is common in FMD, or poor task performance could have had an effect on attention, but this would have been expected to additionally affect the alerting component, and not selectively the executive system.

Is this executive dysfunction a pre-existing and possibly predisposing trait of FMD or is it a secondary effect? A limitation of this study is that it cannot answer this question directly. The ANT would have to be performed in a cohort of recovered patients, or ideally in the same individuals while symptomatic and after recovery. Nevertheless, clinical information and functional imaging data point to it being a secondary effect. Patients do not tend to report pre-existing executive difficulties such as the inability to multitask, concentration difficulties, or even fatigue. Similarly, these symptoms tend to improve during recovery. In the functional imaging literature the executive task of the ANT is reported to activate the anterior cingulate cortex and the prefrontal cortex, particularly the medial frontal cortex (11, 21). The same regions, the ventromedial prefrontal cortex (22–24) and anterior cingulate cortex (25) have previously been reported to show increased activity in patients with functional movement disorders. Importantly, a recent case report demonstrated abnormal medial prefrontal cortex activation in a patient with functional paresis, which normalized after recovery (24). Prefrontal cortical activation has often been interpreted as being due to inhibition of the motor system or of limbic induced abnormal motor patterns (24, 25). Yet an alternative explanation is that it is due to increased self-monitoring and hence increased utilization of the executive function of attention during movement (23). In line with this hypothesis, a PET study in healthy controls showed increased prefrontal cortical activation when a complex finger tapping sequence was learned, disappearance of this activation once the sequence was well practiced and hence performed automatically, and reappearance of this prefrontal cortical activation when participants were asked to attend to what they were doing (26).

The vast majority of movements we make throughout the day are done implicitly, automatically. Yet in functional movement disorders this implicit, low-level control of movement seems to be overridden by explicit, high-level control. As a result, movement becomes unusually cognitively demanding, and draws heavily on limited cognitive resources. Cognitive motor interference with worsening motor function with concomitant cognitive tasks and vice versa is well-established (27, 28). We therefore suggest that the observed executive dysfunction in FMD is secondary to overutilization of the executive network for explicit movement control. In the context of limited attentional resources, executive dysfunction is the likely flip side of distractibility. Because attention is heavily involved in functional movement disorders, its distraction away from the movement leads to symptom improvement. Yet functional movement disorders draw upon limited attentional resources. Tasks that are less attentionally demanding, such as alerting and orienting in space are performed normally. Yet, the remaining attentional resources are insufficient to perform attention heavy executive tasks. Most participants with FMD had an upper limb tremor, and many commented on the presence of their arm unrelated movement disorder during the task. Thus, even though participants were only pressing two keys in a seated position, the functional movement disorders seemed to draw on their attentional resources during the task, thereby preventing them from directing their full attention to it.

Importantly, these findings of impaired executive function provide a so far elusive explanation for many associated, but hitherto seemingly unrelated symptoms. Overutilization of the limited attentional resources by the functional movement disorder, with resulting executive dysfunction for other tasks, can explain why patients with FMD typically complain of cognitive difficulties that are mostly attentional in nature: inability to multitask, concentration difficulties, and the increasingly commonly used term “brain fog,” which is suggestive of inattention. Furthermore, it provides an explanation for the somehow inappropriately termed “huffing and puffing sign” in which relatively simple tasks or movements are performed with extreme slowness and high apparent effort. Finally, fatigue can be explained by a high utilization of attentional resources for even the simplest of movements with an additional subsequent lack of executive resources for other tasks, making both movements and mental activity cognitively harder. A further study could directly study the correlation between the cognitive complaints and/or fatigue with the degree of executive dysfunction in the ANT.

Whether in addition to its impact on cognitive function and fatigue, executive dysfunction negatively impacts functional movement disorders directly, thereby forming a vicious circle, remains an open question. Inability to switch between tasks and shift attention away from the abnormal movements would certainly have a negative impact, since one of the keys to improving functional movement disorders is to be able to shift attention away from the abnormal movements. Thus, executive dysfunction in FMD might merit treatment in its own right.

## Data Availability Statement

Our ethics agreement prevents data being openly available, but individual researchers may request anonymised data.

## Ethics Statement

The studies involving human participants were reviewed and approved by NHS Health Research Authority, London—Bromley Research Ethics Committee (Reference: 16 /LO /1463), and carried out in accordance with the Declaration of Helsinki (29). The patients/participants provided their written informed consent to participate in this study.

## Author Contributions

A-CH designed, conceptualized, programmed the study, recruited the participants, collected, analyzed and interpreted the data, drafted, and revised the manuscript for intellectual content. KB revised the manuscript for intellectual content. ME and PH designed, conceptualized the study, and revised the manuscript for intellectual content. All authors contributed to the article and approved the submitted version.

## Conflict of Interest

The authors declare that the research was conducted in the absence of any commercial or financial relationships that could be construed as a potential conflict of interest.
